# Chicken Glycogen Synthase Kinase 3β Suppresses Innate Immune Responses and Enhances Avian Leukosis Virus Replication in DF-1 Cells

**DOI:** 10.1128/spectrum.05235-22

**Published:** 2023-03-30

**Authors:** Zhouhao Cui, Bei Weng, Yongxiu Yao, Hongxia Shao, Jianqiang Ye, Aijian Qin, Kun Qian

**Affiliations:** a Ministry of Education Key Laboratory for Avian Preventive Medicine, Yangzhou University, Yangzhou, Jiangsu, People’s Republic of China; b Jiangsu Key Laboratory of Preventive Veterinary Medicine, Yangzhou University, Yangzhou, Jiangsu, People’s Republic of China; c The International Joint Laboratory for Cooperation in Agriculture and Agricultural Product Safety, Ministry of Education, Yangzhou University, Yangzhou, Jiangsu, People’s Republic of China; d Jiangsu Co-innovation Center for Prevention and Control of Important Animal Infectious Diseases and Zoonoses, Yangzhou University, Yangzhou, Jiangsu, People’s Republic of China; e The Pirbright Institute & UK-China Centre of Excellence for Research on Avian Diseases, Pirbright, Surrey, United Kingdom; University of Prince Edward Island

**Keywords:** GSK3β, chicken, innate immunity, avian leukosis virus subgroup J

## Abstract

Glycogen synthase kinase 3β (GSK3β) is a widely distributed multifunctional serine/threonine kinase. In mammals, GSK3β regulates important life activities such as proinflammatory response, anti-inflammatory response, immunity, and cancer development. However, the biological functions of chicken GSK3β (chGSK3β) are still unknown. In the present study, the full-length cDNA of chGSK3β was first cloned and analyzed. Absolute quantification of chicken chGSK3β in 1-day-old specific-pathogen-free birds has shown that it is widely expressed in all tissues, with the highest level in brain and the lowest level in pancreas. Overexpression of chGSK3β in DF-1 cells significantly decreased the gene expression levels of interferon beta (IFN-β), IFN regulatory factor 7 (IRF7), Toll-like receptor 3 (TLR3), melanoma differentiation-associated protein 5 (MDA5), MX-1, protein kinase R (PKR), and oligoadenylate synthase-like (OASL), while promoting the replication of avian leukosis virus subgroup J (ALV-J). Conversely, levels of most of the genes detected in this study were increased when chGSK3β expression was knocked down using small interfering RNA (siRNA), which also inhibited the replication of ALV-J. These results suggest that chGSK3β plays an important role in the antiviral innate immune response in DF-1 cells, and it will be valuable to carry out further studies on the biological functions of chGSK3β.

**IMPORTANCE** GSK3β regulates many life activities in mammals. Recent studies revealed that chGSK3β was involved in regulating antiviral innate immunity in DF-1 cells and also could positively regulate ALV-J replication. These results provide new insights into the biofunction of chGSK3β and the virus-host interactions of ALV-J. In addition, this study provides a basis for further research on the function of GSK3 in poultry.

## INTRODUCTION

Glycogen synthase kinase 3 (GSK3) was first identified during the study of glycogen metabolism. During glycogen deposition, GSK3 is the key rate-limiting enzyme in the glycogen formation pathway; it acts as a kinase that promotes phosphorylation of glycogen synthase (1). GSK3 was first cloned in 1990, and it contains two highly homologous but distinct genes (called α and β) (2). GSK3α and GSK3β are encoded from two separate chromosomes, each consisting of 11 exons, and share 98% similarity within their catalytic structural domains (3).

As a widely distributed multifunctional serine/threonine kinase, GSK3 is conserved in eukaryotic evolution (4). It is involved in the regulation of multiple signaling pathways. In the insulin signaling pathway, activated Akt inhibits GSK3 kinase by phosphorylating GSK3α/β, allowing the phosphatase to act on downstream targets such as glycogen synthase, thereby converting the glycogen synthase to an activated state and promoting glycogen synthesis (3). Under nonactivated conditions for the Wnt signaling pathway, a disruption complex consisting of GSK3 coordinates the degradation of β-catenin (3). The role of GSK3 is less clear in the Notch pathway than in the Wnt pathway, and GSK3 has been shown to phosphorylate Notch intracellular domain (NICD) (5). GSK3 can decrease the level of Notch1 protein and reduce the transcriptional activity of Notch1 and Notch2 (6, 7). Additionally, GSK3β-mediated phosphorylation has been reported to stabilize and activate Notch1, possibly by inhibiting the proteasomal degradation of Notch1 (8). In the Hedgehog signaling pathway, GSK3 forms a complex with another two kinases that phosphorylate Gli2/3 and cause Gli2/3 to generate a truncated form, finally translocating to the nucleus and repressing gene transcription. Moreover, in the transforming growth factor β (TGF-β) signaling pathway, GSK3 phosphorylates Smad3, resulting in degradation of the latter (9). Collectively, these signaling pathways are involved in the regulation of metabolism, apoptosis, cell cycle, cell differentiation, embryogenesis, and gene transcription (10, 11), which sheds light on the important role of GSK3 in these cellular processes.

GSK3 also plays a vital role in regulating innate immunity. GSK3β has been reported to promote innate immune response by inhibiting 5′-AMP-activated protein kinase (AMPK) activation (12). Some evidence suggests that the immunomodulatory role of GSK3 lies mainly in affecting type I interferon (IFN) signaling and exerting antiviral effects (13). Martin et al. reported that GSK3 negatively regulates anti-inflammatory cytokine production by macrophages through Toll-like receptor (TLR)-induced activation of the phosphoinositide 3-kinase (PI3K)-Akt signaling pathway (14). Inhibition of GSK3β activity increases the nuclear binding level of cAMP response element-binding protein (CREB), thus promoting the production of interleukin 10 (IL-10) and reducing the production of IL-12 (15). In addition, GSK3 is related to the regulation of proinflammatory and anti-inflammatory responses. Activated GSK3β can promote the activation of NF-κB, which in turn leads to a proinflammatory response. Conversely, MyD88-dependent signaling pathway activation of TLR receptors can promote GSK3β inactivation to lead to an anti-inflammatory response (14, 16–20). Furthermore, infection with some viruses (e.g., human T-cell leukemia virus type 1 [HTLV-1]) causes increased levels of GSK3β phosphorylation and decreased levels of proinflammatory cytokines tumor necrosis factor alpha (TNF-α), IL-8, and monocyte chemotactic protein 1 (MCP-1) (21). Interestingly, the infection of triple transgenic mice with mouse hepatitis virus (MHV) leads to significant increases in GSK3β activity and in the number of cells expressing major histocompatibility complex class II (MHC-II), CD4, and CD8, as well as in levels of proinflammatory cytokines (22), suggesting that different viruses cause different changes in GSK3β activity, but the exact detailed mechanisms still need to be investigated further. Moreover, GSK3 is also relevant to a variety of crucial life activities, such as cell cycle and differentiation, acquired immunity, and carcinogenesis (23).

In contrast to mammals, only one isoform of GSK3β is present in poultry (24). GSK3β has been extensively studied in mammals but has not yet been reported in avian species. In this study, chicken GSK3β (chGSK3β) was cloned and analyzed, and its distribution in various tissues was detected. Then, we detected the effect of chGSK3β on innate immunity and its regulation in avian leukosis virus subgroup J (ALV-J) infection. These results provide novel insights into the role of chGSK3β in regulating the innate immune mechanisms in chicken.

## RESULTS

### Cloning and sequence analysis of chGSK3β.

**FIG 1:**
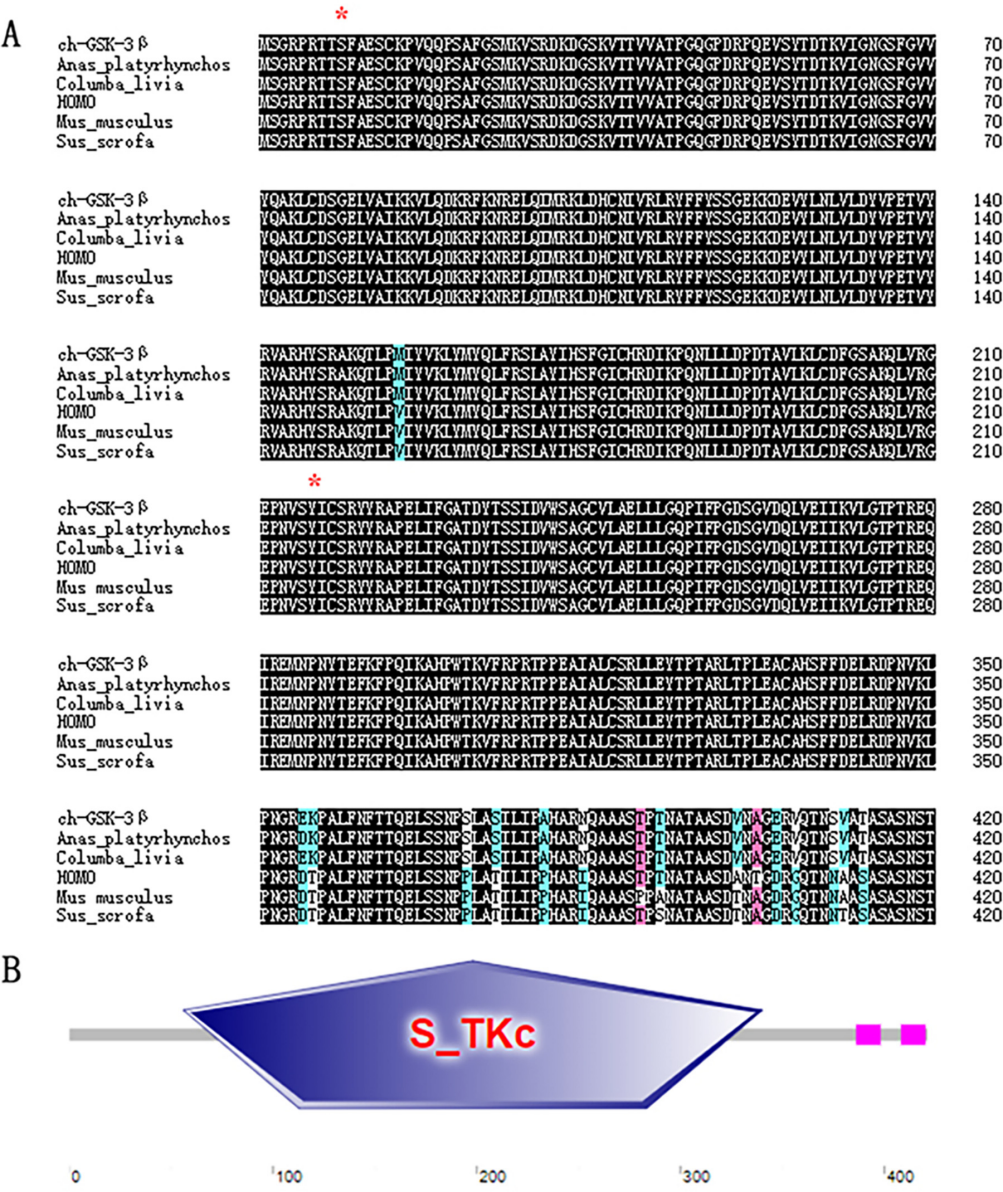
Characteristics of chGSK3β. (A) Amino acid sequence alignment of chicken, human (HOMO), Mus musculus, Sus scrofa, Columba livia, and Anas platyrhynchos GSK3βs using DNAMAN software. Black, sequences conserved across the species; pink, protein sequence homology of ≥75%; light blue, protein sequence homology of ≥50%. Ser9 and Tyr216 are indicated by red asterisks above the sequences. (B) Prediction of protein structural domains using the SMART program. The pink bars indicate two low-complexity domains, located at amino acids 386 to 398 and amino acids 408 to 420 of chGSK3β.

**FIG 2:**
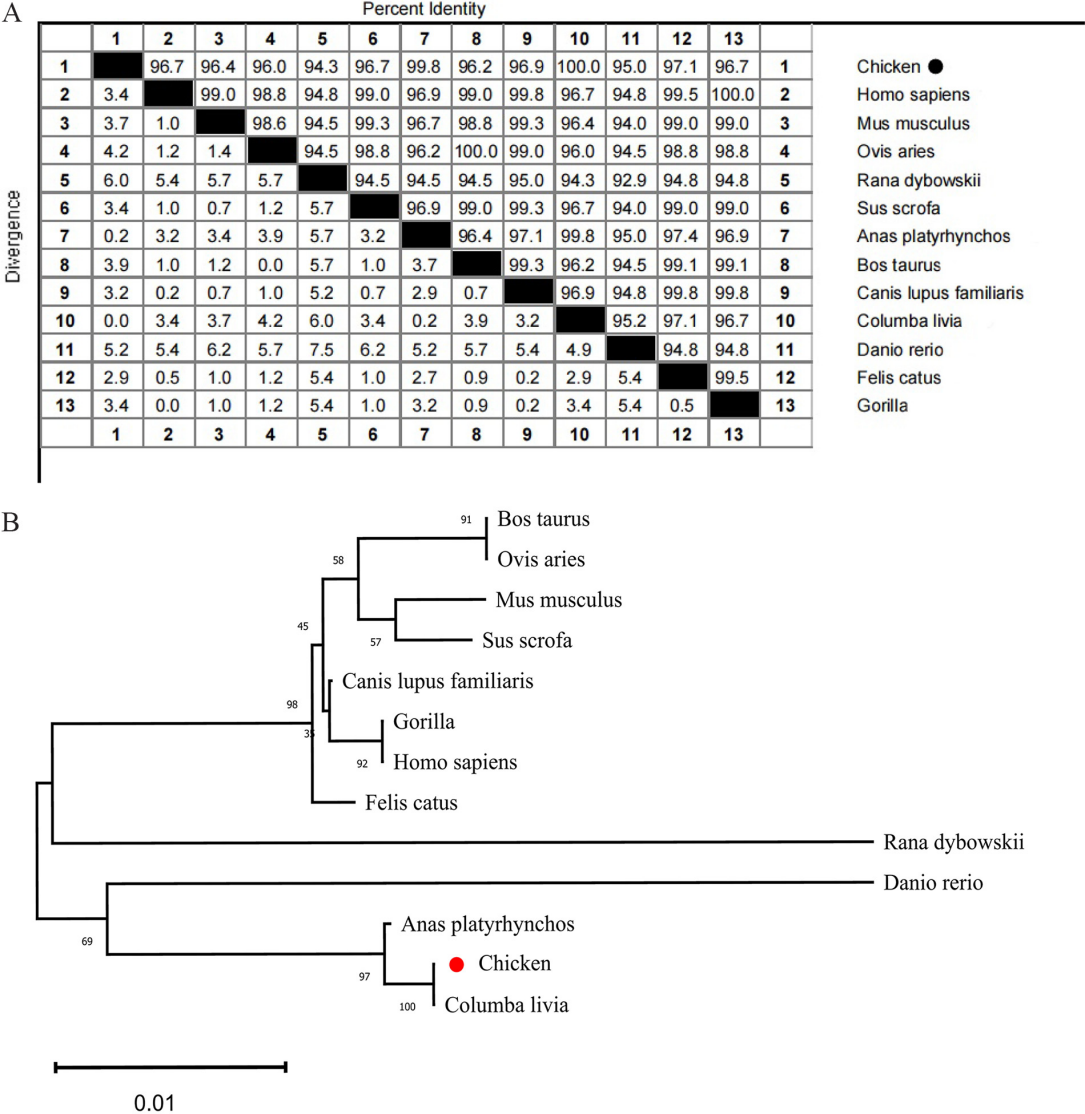
Sequence distance and phylogenetic analyses of chGSK3β. (A) Sequence distance analysis of GSK3β from different species. The analysis was performed with MegAlign software. (B) Phylogenetic tree of the chGSK3 amino acid sequence and other species sequences. The neighbor joining tree was generated using MEGA-X.

The full-length cDNA of chGSK3β (GenBank accession number OP548633.1), with 1,263 bp, encodes 421 amino acids (Fig. 1A). Analysis of the protein structural domain of chGSK3β using SMART software (http://smart.embl-heidelberg.de/) suggested that chGSK3β has a serine/threonine protein kinase (S_TKc) structural domain (amino acids 56 to 340) (Fig. 1B). Multiple-sequence alignment revealed that chGSK3β is relatively conserved in most mammals and birds, and it has the same phosphorylation sites (Ser9 and Tyr216) as other species (Fig. 1A). The amino acid sequence of chGSK3β shares 100%, 95%, 96.7%, 96.4%, and 94.3% identities with the counterparts in wild pigeon, zebrafish, human, mouse, and frog, respectively (Fig. 2A). The phylogenetic tree indicates that chGSK3β branched with birds (Fig. 2B).

### Tissue distribution of chGSK3β in SPF chickens.

**FIG 3:**
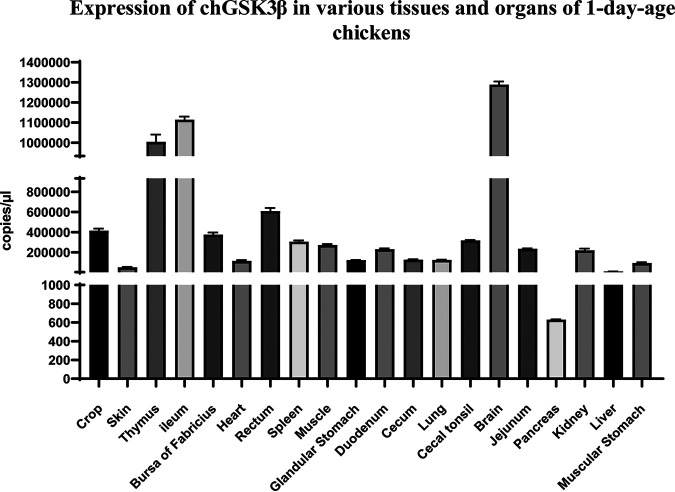
Expression of chGSK3β in various tissues and organs of 1-day-old SPF chickens. An absolute real-time qPCR was used to detect the GSK3β expression levels in different tissues.

The chGSK3β transcript levels in different tissues of 1-day-old specific-pathogen-free (SPF) chickens were measured by quantitative reverse transcription (qRT)-PCR with the RNA extracted from the corresponding tissues. The results showed that chGSK3β levels were relatively higher in brain, ileum, and thymus and lowest in pancreatic tissues (Fig. 3).

### Expression of pCAGGS-chGSK3β-Flag in DF-1 cells.

**FIG 4:**
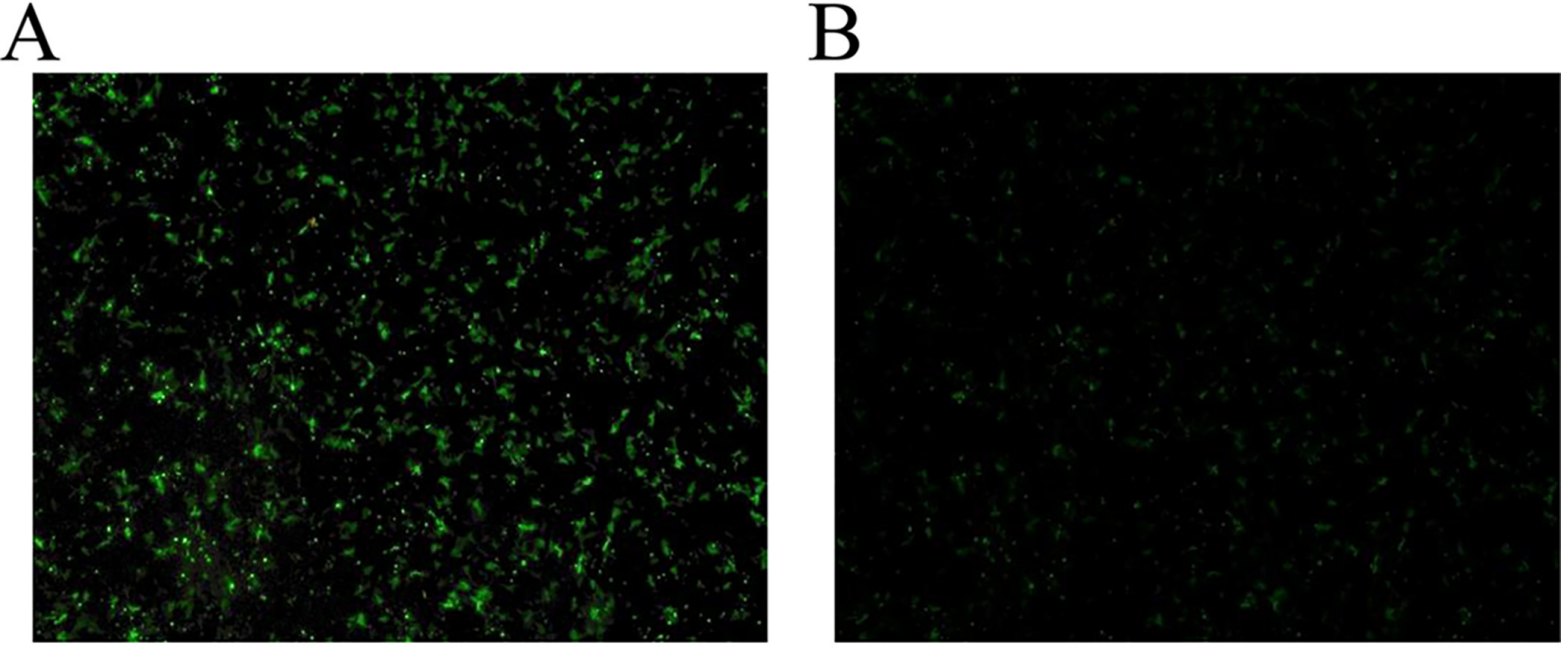
Expression of pCAGGS-chGSK3β-Flag in DF-1 cells. (A) Detection of chGSK3β in pCAGGS-chGSK3β-Flag-transfected DF-1 cells by immunofluorescence assay. (B) Detection of chGSK3β in the pCAGGS empty vector-transfected DF-1 cells by immunofluorescence assay.

Following transfection of pCAGGS-chGSK3β-Flag into DF-1 cells, the expression of chGSK3β was detected by indirect immunofluorescence assay. The result showed that GSK3β was expressed well in DF-1 cells (Fig. 4A and B).

### chGSK3β is involved in the regulation of innate immunity.

**FIG 5:**
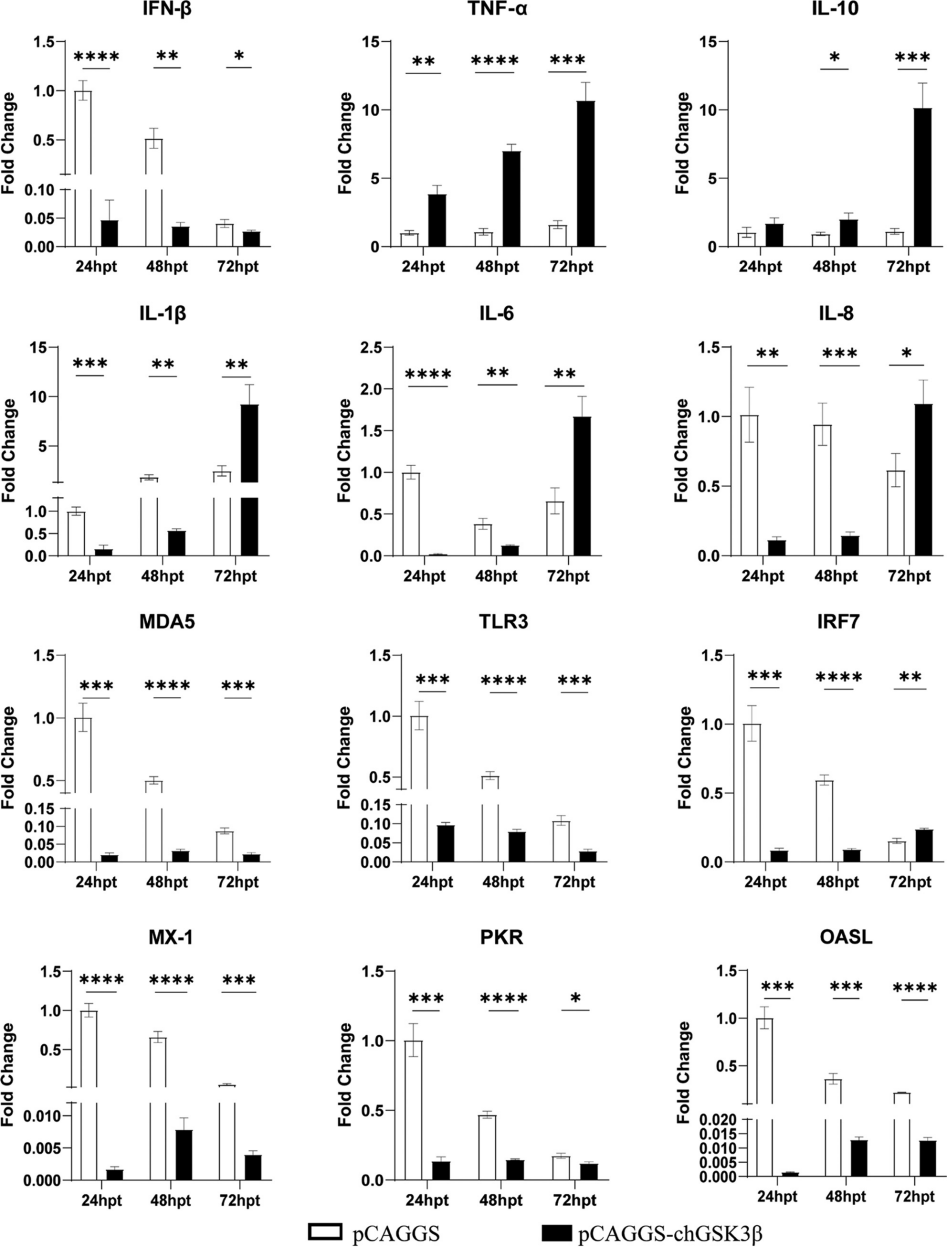
Overexpression of chGSK3β affected the expression of pattern recognition receptors, cytokines, and antiviral molecules in DF-1 cells. The experimental group was DF-1 cells transfected with pCAGGS-chGSK3β-Flag, and the control group was DF-1 cells transfected with empty vector. Total cellular RNA was collected at 24, 48, and 72 hpt, reverse transcribed, and analyzed by qRT-PCR for pattern recognition receptors (TLR3 and MDA5), proinflammatory cytokines (IL-1β, IL-6, IL-8, and TNF-α), anti-inflammatory cytokines (IL-10), IFN-β, IRFs (IRF3 and IRF7), and antiviral molecules (MX-1 and PKR). The relative expression of mRNAs was calculated using the 2^−ΔΔ^*^CT^* method, with the 18S rRNA gene as the internal reference gene. *, *P* < 0.05; **, *P* < 0.01; ***, *P* < 0.001; ****, *P* < 0.0001. These experiments were performed independently at least three times, with similar results.

**FIG 6:**
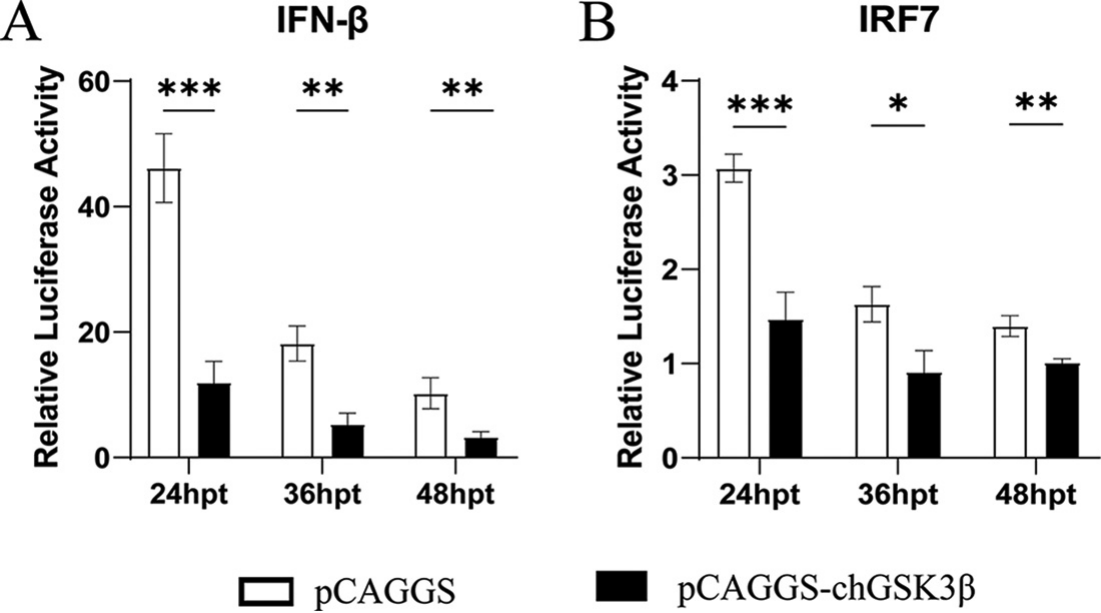
Overexpression of chGSK3β significantly downregulated the promoter activity of IFN-β and IRF7 in DF-1 cells. We used a dual luciferase reporter assay to study the effect of chGSK3β on the IFN-β signaling pathway. The pCAGGS-chGSK3β-Flag or empty vector was cotransfected with reporter gene plasmids pGL3-IFN-β (A) or pGL3-IRF7 (B) together with pRL-TK plasmid. Cells were harvested at 24, 48, and 72 hpt for dual luciferase activity assays. Differences were assessed using the *t* test. *, *P* < 0.05; **, *P* < 0.01; ***, *P* < 0.001. These experiments were performed independently at least three times, with similar results.

To investigate the role of chGSK3β in the chicken innate immune response, pCAGGS-chGSK3β-Flag or empty vector was transfected into DF-1 cells. The total cellular RNA was extracted from cells collected at the corresponding time and reverse transcribed for subsequent experiments (Fig. 5). Compared with the control group, the mRNA expression of IFN-β and IFN regulatory factor 7 (IRF7) was significantly decreased at 24 h and 48 h in the overexpression group. Levels of proinflammatory factors (IL-1β, IL-6, and IL-8) were also significantly decreased at 24 h and 48 h but increased at 72 h. The levels of the pattern recognition receptors (melanoma differentiation-associated protein 5 [MDA5] and TLR3) and antiviral molecules (protein kinase R [PKR] and MX-1) continuously decreased, while the expression of TNF-α and IL-10 was significantly elevated. Dual luciferase reporter assays also demonstrated that overexpression of chGSK3β significantly downregulated the promoter activity of IFN-β and IRF7 (Fig. 6). Overexpression of chGSK3β in DF-1 cells had no significant effect on the NF-κB promoter activity (data not shown).

**FIG 7:**
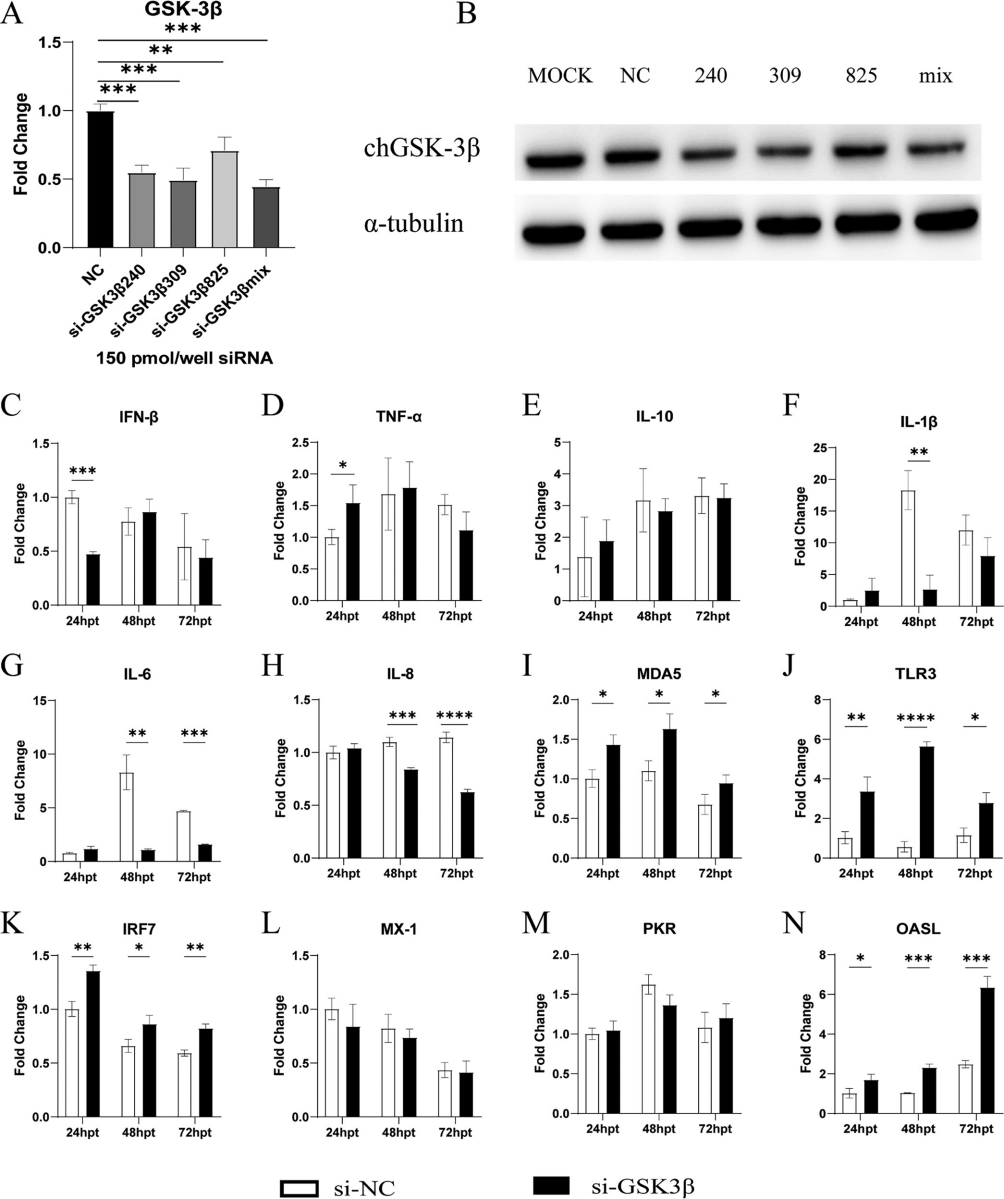
Inhibition of chGSK3β significantly affected the expression of pattern recognition receptors, cytokines, and antiviral molecules in DF-1 cells. (A) DF-1 cells in 12-well plates were transfected with siRNAs targeting chGSK3β or a control vector. After transfection for 24 h, total cellular RNA was extracted, and the chGSK3β gene expression level was measured by qRT-PCR. (B) The chGSK3β protein expression level was measured by Western blotting. (C to N) DF-1 cells transfected with siRNAs targeting chGSK3β or a control vector were collected at 24, 48, and 72 hpt and analyzed for mRNA expression levels of TLR3, MDA5, proinflammatory cytokines (IL-1β, IL-6, IL-8, and TNF-α), anti-inflammatory cytokines (IL-10), IFN-β, IRF7, and antiviral molecules (MX-1, PKR, and OASL) by qRT-PCR. The relative expression of the transcripts was calculated using the 2^−ΔΔ^*^CT^* method, with the 18S rRNA gene as an internal reference gene. Data were expressed as mean ± SD from three independent experiments. *, *P* < 0.05; **, *P* < 0.01; ***, *P* < 0.001; ****, *P* < 0.0001.

After the inhibition of chGSK3β expression by small interfering RNA (siRNA), the mRNA expression levels of proinflammatory factors (IL-1β, IL-6, and IL-8) decreased to different degrees, and the mRNA expression of MDA5, TLR3, IRF7, and antiviral molecules (oligoadenylate synthase-like [OASL]) increased significantly in the chGSK3β-inhibited group, compared with the control group (Fig. 7). These results suggested that chGSK3β was involved and regulated chicken innate immunity.

### chGSK3β positively regulates the replication of ALV-J.

**FIG 8:**
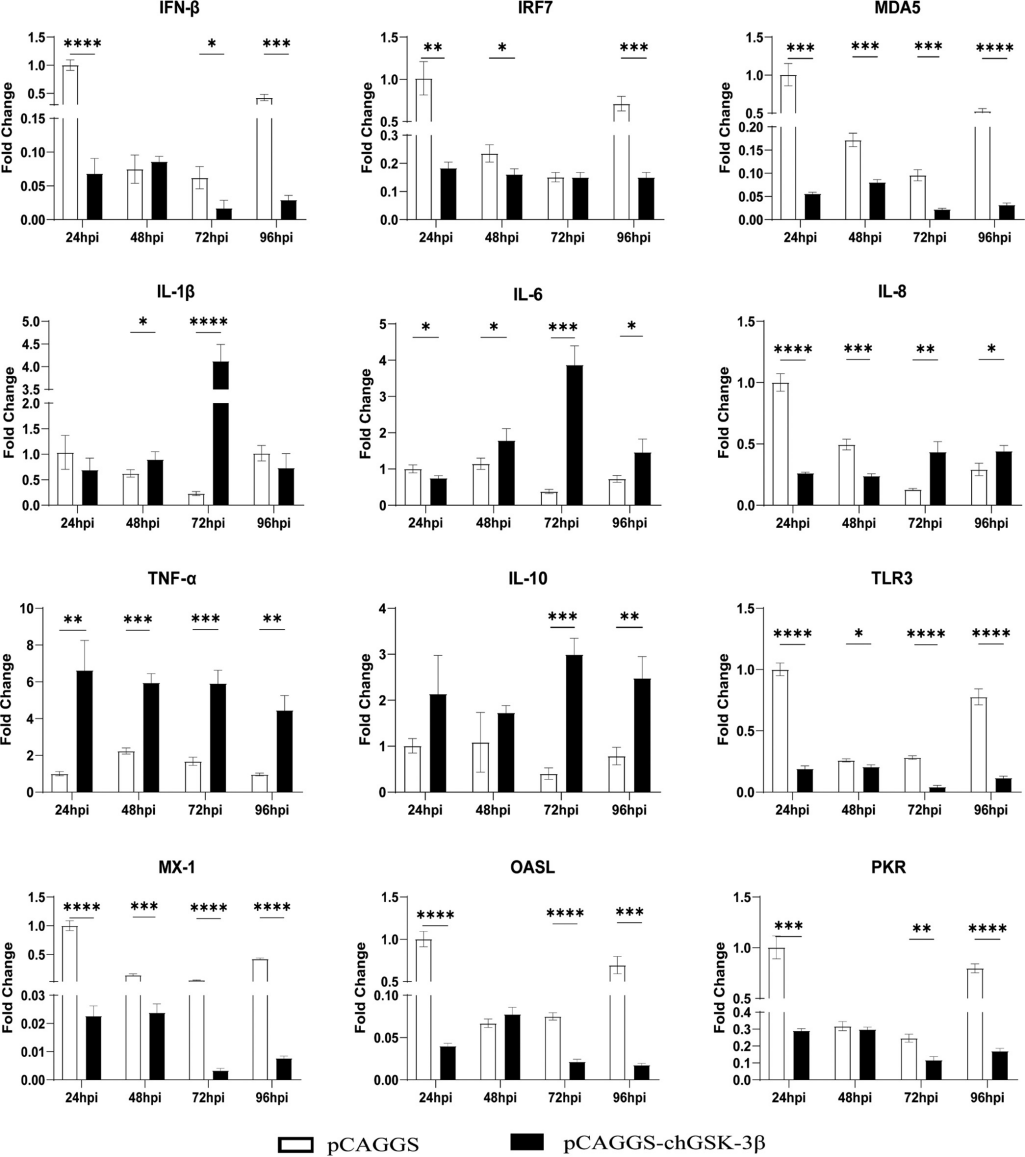
Overexpression of chGSK3β in DF-1 cells regulates the expression of pattern recognition receptors and cytokines after ALV-J infection. The DF-1 cells were transfected with pCAGGS-chGSK3β-Flag, and empty vector served as a control. Twenty-four hours after transfection, cells were infected with ALV-J (MOI of 1.0). Total cellular RNA was extracted from the cells collected at different time points, and mRNA expression levels of the selected genes were measured by qRT-PCR for TLR3, MDA5, proinflammatory cytokines (IL-1β, IL-6, IL-8, and TNF-α), anti-inflammatory cytokines (IL-10), IFN-β, IRF7, and antiviral molecules (MX-1, OASL, and PKR). The relative expression of mRNAs was calculated using the 2^−ΔΔ^*^CT^* method, with the 18S rRNA gene as the internal reference gene. Data were expressed as mean ± SD from three independent experiments. *, *P* < 0.05; **, *P* < 0.01; ***, *P* < 0.001; ****, *P* < 0.0001.

**FIG 9:**
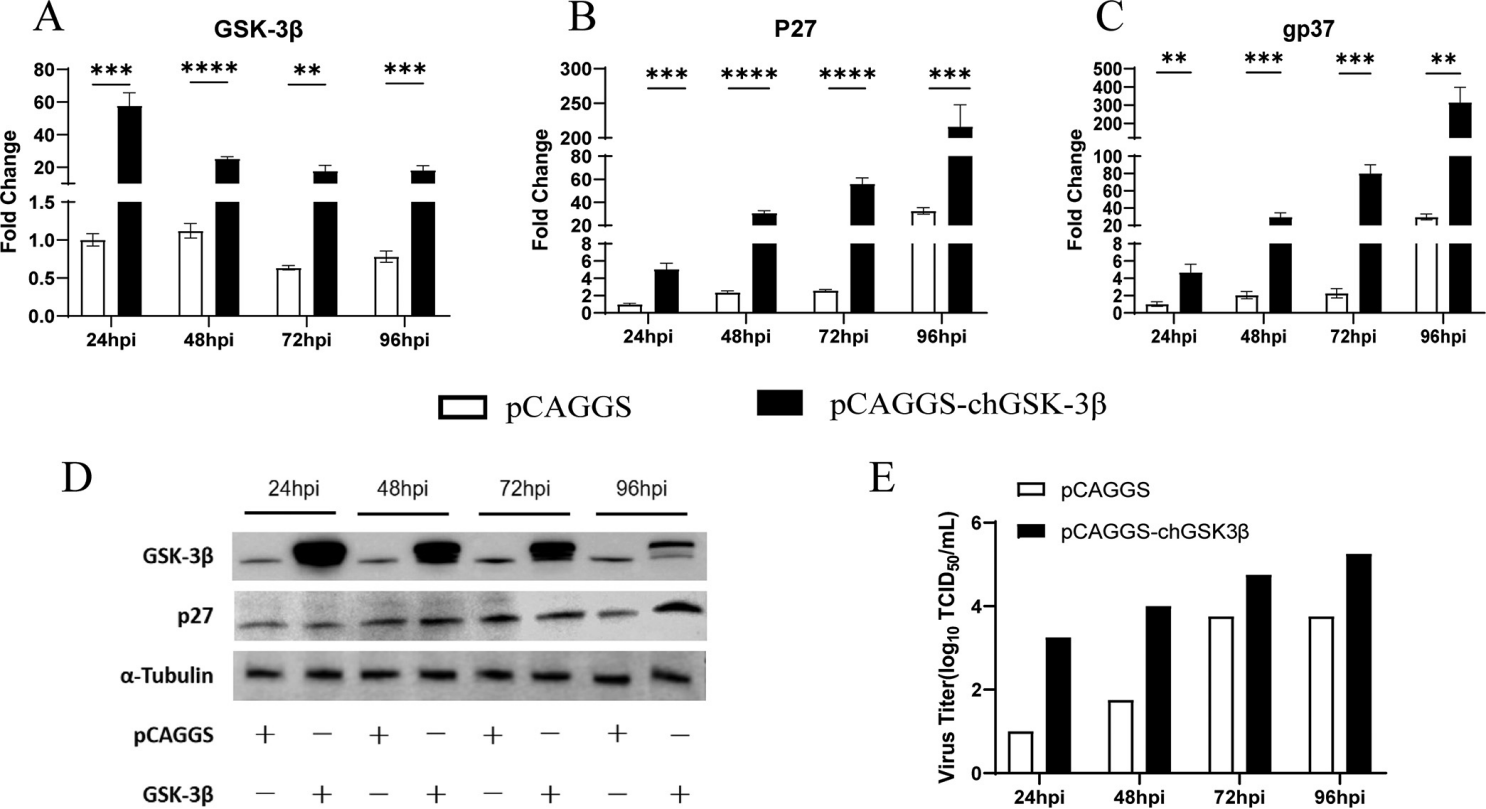
Overexpression of chGSK3β promotes ALV-J replication in DF-1 cells. The experimental group was DF-1 cells transfected with pCAGGS-chGSK3β-Flag, and the control group was DF-1 cells transfected with empty vector. Twenty-four hours after transfection, cells were infected with ALV-J (MOI of 1.0). (A to C) Total cellular RNA was extracted from cells collected at different time points, and the mRNA expression levels of chGSK3β (A) and ALV-J genes p27 (B) and gp37 (C) were measured by qRT-PCR. The relative expression of mRNAs was calculated using the 2^−ΔΔ^*^CT^* method, with the 18S rRNA gene as the internal reference gene. Data were expressed as mean ± SD from three independent experiments. **, *P* < 0.01; ***, *P* < 0.001; ****, *P* < 0.0001. (D) The protein levels of virally encoded P27 and chGSK3β in cell were measured by immunoblotting. (E) The virus titer in the cell supernatants was measured by TCID_50_ assays.

**FIG 10:**
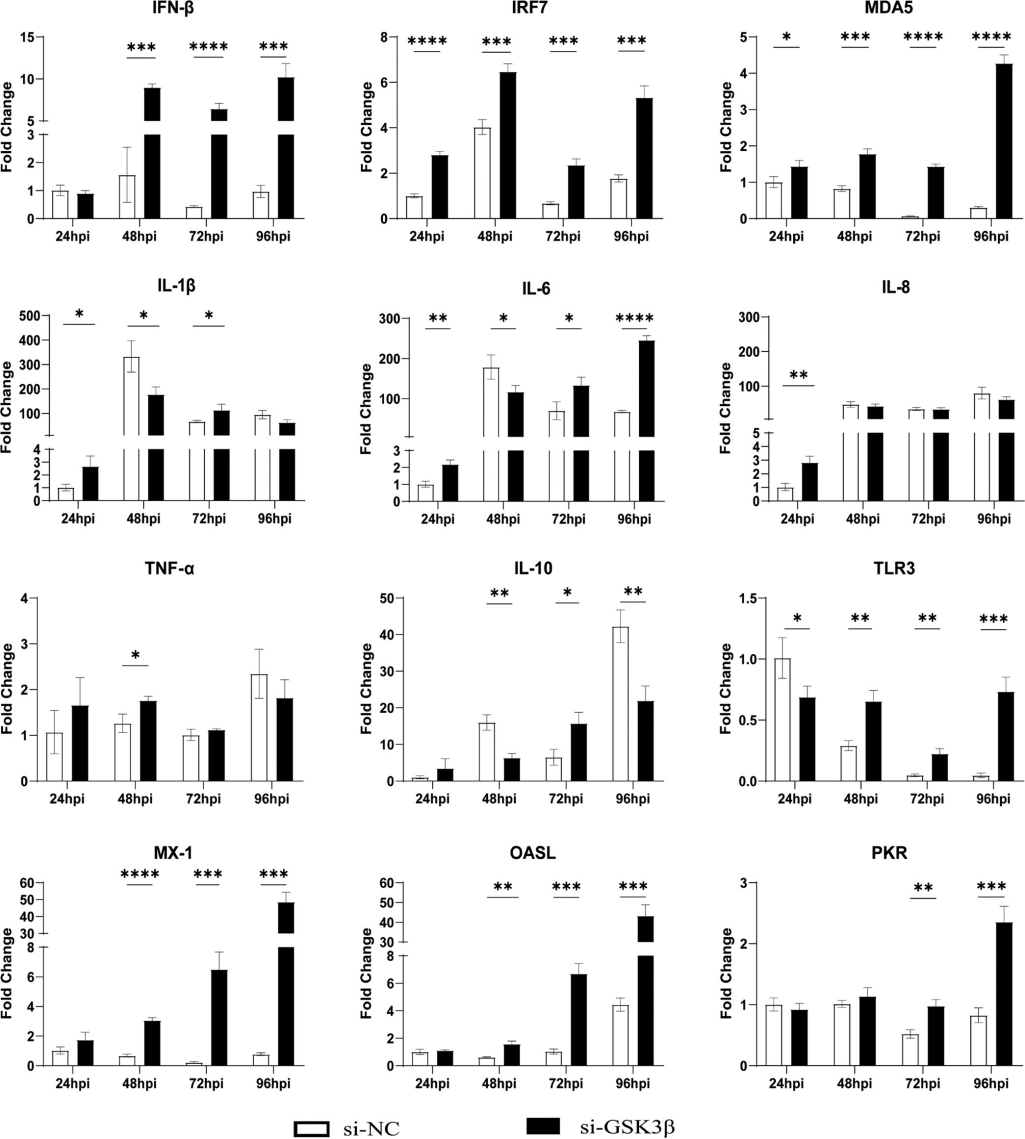
ALV-J infection regulates the expression of pattern recognition receptors and cytokines after chGSK3β inhibition in DF-1 cells. The experimental group was DF-1 cells transfected with GSK3β siRNA, and the control group was DF-1 cells transfected with siNC. Twenty-four hours after transfection, cells were infected with ALV-J (MOI of 1.0). Total cellular RNA was extracted from cells collected at different time points, and transcript levels were measured by qRT-PCR for pattern recognition receptors (TLR3 and MDA5), proinflammatory cytokines (IL-1β, IL-6, IL-8, and TNF-α), anti-inflammatory cytokines (IL-10), IFN-β, IRF7, and antiviral molecules (MX-1, OASL, and PKR). The relative expression of gene mRNAs was calculated using the 2^−ΔΔ^*^CT^* method, with the 18S rRNA gene as the internal reference gene. Data were expressed as mean ± SD from three independent experiments. *, *P* < 0.05; **, *P* < 0.01; ***, *P* < 0.001; ****, *P* < 0.0001.

**FIG 11:**
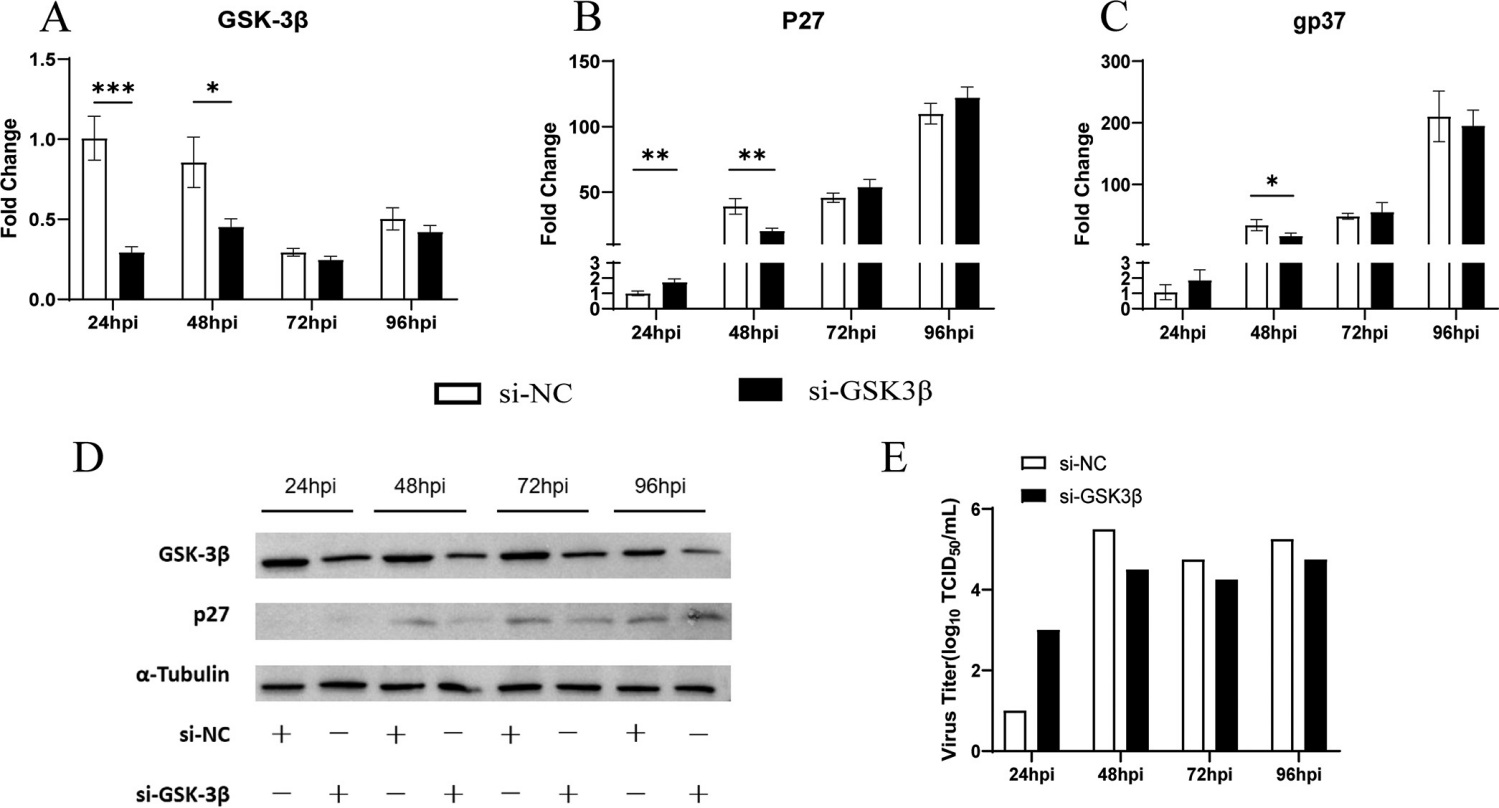
Inhibition of chGSK3β suppressed viral replication of ALV-J in DF-1 cells. The experimental group was DF-1 cells transfected with GSK3β siRNA, and the control group was DF-1 cells transfected with siNC. Twenty-four hours after transfection, the cells were infected with ALV-J (MOI of 1.0). (A to C) Total cellular RNA was extracted from cells collected at different time points, and the mRNA expression levels of chGSK3β (A) and ALV-J genes p27 (B) and gp37 (C) were measured by qRT-PCR. The relative expression of gene mRNAs was calculated using the 2^−ΔΔ^*^CT^* method, with the 18S rRNA gene as the internal reference gene. Data were expressed as mean ± SD from three independent experiments. *, *P* < 0.05; **, *P* < 0.01; ***, *P* < 0.001. (D) The protein levels of virally encoded P27 and chGSK3β in cells were measured by immunoblotting. (E) The virus titer in the cell supernatants was measured by TCID_50_ assays.

To explore the effect of chGSK3β on virus replication of ALV-J, chGSK3β was overexpressed or inhibited in ALV-J-infected cells to detect the change of virus replication and the expression levels of innate immune molecules. The results showed that the mRNA expression of IFN-β, IRF7, MDA5, TLR3, PKR, and MX-1 was significantly decreased in the overexpression group after viral infection, compared with the control group. The mRNA levels of IL-1β, IL-6, and IL-8 showed a trend of decreases and then increases, while the mRNA levels of TNF-α and IL-10 significantly increased after viral infection (Fig. 8). The gene expression levels of p27 and gp37 of ALV-J were significantly increased in DF-1 cells transfected with chGSK3β (Fig. 9B and C). The expression level of p27 protein was also increased (Fig. 9D). The titer of the virus in the cell supernatant increased at each time point, as demonstrated by the 50% tissue culture infective dose (TCID_50_) assay (Fig. 9E). These results indicated that overexpression of chGSK3β promoted the replication and proliferation of ALV-J. After inhibition of chGSK3β by siRNA, the mRNA expression of IFN-β, IRF7, MDA5, TLR3, PKR, MX-1, and OASL were significantly upregulated, compared with the control group (Fig. 10), and the gene expression, protein expression, and proliferation of the ALV-J were significantly decreased (Fig. 11), which indicated that the inhibition of chGSK3β suppressed the replication of ALV-J in DF-1 cells. All of these results demonstrated that chGSK3β had the biological function of promoting the replication of ALV-J.

## DISCUSSION

GSK3β is an important multifunctional protein involved in the regulation of multiple signaling pathways, innate immunity, and inflammatory responses in mammals (3, 16, 25). However, the function of chGSK3β has not yet been investigated. In this study, the complete chGSK3β cDNA (GenBank accession number OP548633) was cloned for the first time; it is 1,263 bp long and encodes 421 amino acids. Results from BLAST analysis revealed that the chGSK3β gene is conserved in a variety of species. It shares the Ser9 and Tyr216 phosphorylation sites with other species, which affects the biological function of GSK3. To investigate the tissue distribution of chGSK3β, the abundance of the chGSK3β gene in different tissues was analyzed. The chGSK3β gene expression was found to be widely distributed in different tissues. The highest expression level of chGSK3β is in brain, which is consistent with findings in mammals (26).

To further explore whether chGSK3β is involved in avian innate immunity, the chGSK3β was overexpressed in DF-1 cells. We found that the gene expression of IFN-β, IRF7, TLR3, MDA5, MX-1, and PKR was significantly decreased after overexpression of chGSK3β in DF-1 cells. However, the expression of TNF-α was significantly increased, in support of the previous report that GSK3 positively regulates TNF-α production to modulate the innate immune response (27). Notably, previous reports showed that GSK3β could regulate IL-10 expression negatively (28–30), whereas chGSK3β significantly promoted IL-10 expression levels in our study, as shown in Fig. 5; this may be due to the different mechanisms of IL-10 regulation by GSK3β in different cells and species. Moreover, the results in Fig. 6 revealed that overexpression of chGSK3β could inhibit IFN-β promoter activity and reduce IFN-β expression also, which suggested that chGSK3β might be involved in IFN-associated immunity.

Many studies have reported that GSK3 is associated with regulating signaling pathways to affect viral replication. For example, the replication of severe acute respiratory syndrome coronavirus (SARS-CoV) was shown to be dependent on GSK3-mediated phosphorylation of nucleocapsid protein (N), which is an essential process for viral replication (31). Our previous studies demonstrated that GSK3 inhibitors significantly increased the replication of ALV-J in CEF cells (32). The activation of the PI3K/Akt signaling pathway exerted an inhibitory effect on porcine epidemic diarrhea virus (PEDV) proliferation by inhibiting the activity of GSK3. It was also reported that a specific inhibitor of GSK3 could significantly increase the proliferation of PEDV but limit Tat-mediated HIV-1 replication (33). In addition, GSK3β affects hepatitis C virus (HCV) particle maturation and release and promotes the replication of influenza viruses (34, 35). In this study, overexpression of chGSK3β gave rise to significant decreases in the gene expression levels of host antiviral molecules and increased the ALV-J replication, while the virus replication was suppressed significantly after chGSK3β inhibition by siRNA, which suggests that chGSK3β can positively regulate the ALV-J replication in DF-1 cells. It is noteworthy that ALV-J infected cells did not induce any IFN-β expression in DF-1 cells (data not shown). However, knockdown of chGSK3β resulted in a significant increase in IFN-β expression in virus-infected DF-1 cells (Fig. 10), which suggests that ALV-J-induced immunosuppression might be associated with chGSK3β. In previous reports, overexpression of GSK3β was reported to enhance the activation of IRF3 and the transcription of IFN-β by Sendai virus (SeV), and this effect is produced by GSK3β by causing oligomerization and phosphorylation of TANK-binding kinase 1 (TBK1) (36). It was also reported that GSK3β can interact directly with TBK1 (37) to promote TBK1 phosphorylation (38) and that overexpression of TBK1 in DEF cells causes activation of GSK3β (39). In this study, whether the mechanism of chGSK3β affecting ALV-J replication is also related to TBK1 needs to be clarified by further studies.

In summary, the chGSK3β gene was cloned and analyzed for the first time. We found that chGSK3β has high homology with genes from other birds and mammals and is widely expressed in different tissues. The results of biofunctional study reveal that chGSK3β is involved in regulating antiviral innate immunity in DF-1 cells and also can positively regulate ALV-J replication. In addition, the role of GSK3 in immunosuppression induced by ALV-J infection deserves further study.

## MATERIALS AND METHODS

### Animals, cells, and viruses.

SPF chickens were purchased from Lihua Company (China). DF-1 cells were incubated at 37°C in a 5% CO_2_ incubator. The ALV-J strain JS09GY3 (GenBank accession number GU982308.1) was maintained at the Ministry of Education Key Laboratory for Avian Preventive Medicine, Yangzhou University.

### Molecular cloning of chGSK3β.

To amplify chGSK3β, primers chGSK3β F and chGSK3β R (Table 1) were designed based on the predicted chGSK3β sequence in GenBank XM_040660411.2. Total RNA was extracted from HD11 cells by the method of the FastPure cell/tissue total RNA isolation kit v2 (Vazyme, Nanjing, China) and reverse transcribed into cDNA using the HiScript III RT SuperMix for quantitative PCR (qPCR) (Vazyme), and PCR was performed to amplify the target sequence. The pCAGGS-chGSK3β-Flag vector was constructed by inserting the PCR product of chGSK3β into a pCAGGS empty vector digested by XhoI and NotI.

**TABLE 1:** Sequences of primers used in this study

Primer name	Sequence (5′ to 3′)
chGSK3β-F	GCGCGGCCGCATGTCCGGGCGGCCCAGGA
chGSK3β-R	GCCTCGAGCTTATCGTCGTCATCCTTGTAATCGGTGGAGTTGGAGGCTGATGCTGTA
qchGSK3β-F	CTACAGGGCACCAGAGTTGA
qchGSK3β-R	CCACTGTCACCTGGGAAGAT
qIFN-β-F	GCCCACACACTCCAAAACACTG
qIFN-β-R	TTGATGCTGAGGTGAGCGTTG
qTNF-α-F	TGTGTATGTGCAGCAACCCGTAGT
qTNF-α-R	GGCATTGCAATTTGGACAGAAGT
qIL-10-F	ACTGAGTTAACCCACTGCCT
qIL-10-R	CTCCTCTTCTCGCAGGTGAA
qIL-1β-F	TAGATGTCGTGTGTGATGAG
qIL-1β-R	GTAGAAGATGAAGCGGGTC
qIL-6-F	CGACGAGGAGAAATGC
qIL-6-R	TGGCGAGGAGGGAT
qIL-8-F	ACGCTGGTAAAGATGGGGAA
qIL-8-R	GCACACCTCTCTTCCATCC
qMDA5-F	ATGAAATTGCTAGGTGCAGGCCC
qMDA5-R	TCTTGGACACGCGAATGGCCTTA
qTLR3-F	GCACCTGTGAAAGCATTG
qTLR3-R	TAGGCGGGGTGTTACAAATG
qIRF7-F	GAGCCTCCTCCCTCAACAGT
qIRF7-R	AGGGACACAGGAAGGGAGTG
qMX-1-F	AATAAGGCTACTATCCCACA
qMX-1-R	GTGTACTTTTGGAGTTCCTT
qPKR-F	ATCTCCTCTACCTGCGGATG
qPKR-R	GGGTCTCCGGTACGGTTTAT
qOASL-F	GAGATAGAGAAGGAGTGGTG
qOASL-R	GTAGACTGTGGTCTTGTTAC
q18S-F	TCAGATACCGTCGTAGTTCC
q18S-R	TTCCGTCAATTCCTTTAAGTT
qp27-F	CCGGGGAATTGGTTGCTAT
qp27-R	AGTCAATGATCACCGGAGCC
qgp37-F	TGCGTGCGTGGTATTATTTC
qgp37-R	AATGGTGAGGTCGCTGACTGT

### Biological analysis of chGSK3β.

The amino acid sequence was deduced from the nucleotide sequence of the gene and analyzed by DNAMAN. The structural domain of chGSK3β was identified by the SMART program. Homology analysis of amino acid sequences was performed using MegAlign (DNAstar, USA). Phylogenetic trees of 14 different species, including birds, fish, mammals, and arthropods, based on chGSK3β were constructed using MEGA-X.

### Tissue sample collection and real-time qPCR.

Three 1-day-old SPF white leghorn laying hens were used for collection of tissue samples, including the skin, thymus, ileum, bursa of Fabricius, heart, rectum, spleen, muscle, glandular stomach, duodenum, cecum, cecal tonsil, lung, brain, jejunum, pancreas, liver, and muscular stomach. All of the tissue collection procedures were reviewed and approved by the Animal Care Committee of Yangzhou University. Total RNA was extracted from these tissues and reverse transcribed into cDNA. In this experiment, absolute real-time qPCR was used to detect the expression level of GSK3β in each tissue. Three replications were performed for each group. The constructed pCAGGS-chGSK3β-Flag vector was used for 10-fold serial dilutions, and eight concentrations from 1:10^2^ to 1:10^9^ were used as the templates for the standard curve. The real-time qPCR was performed and the reactions were carried out using the ChamQ SYBR qPCR Master Mix (Vazyme) in an ABI 7500 system. The primers are presented in Table 1.

### Western blot analysis.

To verify the expression of chGSK3β-Flag in cells, DF-1 cells were transfected with pCAGGS-chGSK3β-Flag or pCAGGS empty vector using Lipofectamine 3000 reagent (Invitrogen, Carlsbad, CA, USA). The cells were lysed with a cell lysis buffer containing protease inhibitor cocktail (Beyotime, Shanghai, China), and the protein concentrations were determined with a bicinchoninic acid (BCA) protein assay kit (Vazyme). The target proteins were electrotransferred to nitrocellulose membranes (Sigma, Shanghai, China) after SDS-PAGE. The primary antibody was anti-GSK3β monoclonal antibody (MAb) (Cell Signaling Technology, Shanghai, China), and the secondary antibody was horseradish peroxidase (HRP)-conjugated goat anti-mouse IgG (Jackson ImmunoResearch Laboratories, USA).

### Dual luciferase reporter assay.

The dual luciferase reporter assay kit (Vazyme) assisted in detecting gene regulation by measuring the fluorescence intensity of luciferin substrate in response to luciferase expression after transfection of cells with a reporter plasmid. DF-1 cells seeded in 12-well plates were grown to 90% confluence and cotransfected with pCAGGS-chGSK3β-Flag, reporter plasmids (pGL3-IFN-β, pGL3-IRF7, and pGL3-NF-κB), and control plasmid (pRL-TK). The cells were lysed at the indicated time points for dual luciferase reporter assays with a dual-specific luciferase assay kit (Promega, Shanghai, China). Three replications were conducted for each sample.

### Immunofluorescence staining.

The cells were fixed with 4% formaldehyde in phosphate-buffered saline (PBS) for 20 min, permeabilized with 0.25% Triton X-100 for 10 min, and blocked with 2% bovine serum albumin in PBS for 30 min. Then, the cells were incubated with anti-GSK3β MAb (Cell Signaling Technology) in PBS for 45 min at room temperature. The cells were then washed in PBS and incubated with fluorescein isothiocyanate (FITC)-conjugated goat anti-mouse IgG (Jackson ImmunoResearch Laboratories) for 40 min. The cells were visualized with a fluorescence microscope (Olympus IX65).

### RNA interference assay.

Three siRNAs targeting chGSK3β were synthesized by Suzhou Jima Pharmaceutical Technology. Then 150 pmol siRNAs or the negative control (siRNA for the negative control [siNC]) were transfected into DF-1 cells in 12-well plates using Lipofectamine reagents (Invitrogen, Shanghai, China). Total RNA was extracted for real-time PCR at 48 h posttransfection (hpt). The siRNA sequences are presented in Table 2.

**TABLE 2:** siRNA sequences

siRNA primer name	Sequence (5′ to 3′)	Position
si-NC (sense)	UUCUCCGAACGUGUCACGUTT	
si-NC (antisense)	ACGUGACACGUUCGGAGAATT	
si-chGSK3β-240 (sense)	GCUUGUGGCCAUUAAGAAATT	240
si-chGSK3β-240 (antisense)	UUUCUUAAUGGCCACAAGCTT	
si-chGSK3β-309 (sense)	GCUGGAUCAUUGUAACAUUTT	309
si-chGSK3β-309 (antisense)	AAUGUUACAAUGAUCCAGCTT	
si-chGSK3β-825 (sense)	GCCUACAAGGGAGCAAAUUTT	825
si-chGSK3β-825 (antisense)	AAUUUGCUCCCUUGUAGGCTT	

### Viral infection.

After transfection with pCAGGS-chGSK3β-Flag and pCAGGS vector control or si-GSK3β and siNC for 24 h, the DF-1 cells were infected with the JS09GY3 strain at a multiplicity of infection (MOI) of 1.0. The cells were collected at different time points for real-time qPCR and Western blotting, and the culture supernatant was harvested for virus titer determination with the TCID_50_ assay.

### Data analysis.

The data were expressed as mean ± standard deviations (SD). The significance of the variability between the trials was analyzed using GraphPad Prism v8.0 software. Differences in data were evaluated with the Student *t* test.

### Data availability.

The full-length cDNA of chGSK3β was deposited in GenBank under accession number OP548633.1.
